# Severe preeclampsia is associated with a higher relative abundance of *Prevotella bivia* in the vaginal microbiota

**DOI:** 10.1038/s41598-020-75534-3

**Published:** 2020-10-26

**Authors:** Chia-Ying Lin, Chiao-Yun Lin, Yuan-Ming Yeh, Lan-Yan Yang, Yun-Shien Lee, Angel Chao, Chia-Yin Chin, An-Shine Chao, Chia-Yu Yang

**Affiliations:** 1 Department of Pediatrics, Chang Gung Memorial Hospital Linkou Medical Center and Chang Gung University College of Medicine, Taoyuan, Taiwan; 2 Department of Obstetrics and Gynecology, Chang Gung Memorial Hospital Linkou Medical Center and Chang Gung University College of Medicine, Taoyuan, Taiwan; 3 Gynecologic Cancer Research Center, Chang Gung Memorial Hospital, Taoyuan, Taiwan; 4 Genomic Medicine Core Laboratory, Chang Gung Memorial Hospital, Taoyuan, Taiwan; 5 Biostatistics Unit, Clinical Trial Center, Chang Gung Memorial Hospital Linkou Medical Center, Taoyuan, Taiwan; 6 Department of Biotechnology, Ming Chuan University, Taoyuan, Taiwan; 7 Molecular Medicine Research Center, Chang Gung University, Taoyuan, Taiwan; 8 Department of Obstetrics and Gynecology, New Taipei Municipal Tu Cheng Hospital, 6, Sec. 2, Jincheng Road, Tu Cheng District, New Taipei City, 236 Taiwan; 9 Department of Microbiology and Immunology, College of Medicine, Chang Gung University, Taoyuan, Taiwan; 10 Department of Otolaryngology—Head and Neck Surgery, Chang Gung Memorial Hospital, Taoyuan, Taiwan

**Keywords:** Microbiology, Health care

## Abstract

We sought to compare the vaginal microbiota profiles of Taiwanese women with severe preeclampsia (SPE) and normotensive control pregnancies. In a discovery cohort, vaginal swab samples and paired blood specimens were simultaneously obtained at the time of caesarean delivery from 30 women with SPE and 30 controls. The composition of vaginal microbiota was characterised by 16S ribosomal RNA gene sequencing of the V3–V4 region. Results were subsequently validated by real-time qPCR. We sought confirmation of our findings in an expanded cohort consisting of 58 women with SPE and 55 controls. In both the discovery and confirmation cohorts, women with SPE had higher relative abundance of *Prevotella bivia* in their vaginal microbial community (*P* = 0.006 and 0.011, respectively). Plasma levels of tumour necrosis factor alpha (TNF-α) were higher when compared with controls (*P* = 0.031) in the confirmation cohort. Three variables (vaginal *Prevotella bivia*, plasma TNF-α, and body mass index [BMI]) were included in a prediction panel for SPE. Of these, BMI was the most predictive variable. The area under the curve (AUC) of predicted probability values for the three-variable panel revealed that it can discriminate between SPE and normotensive pregnancies with good accuracy (AUC = 0.797, *P* < 0.001). We conclude that enrichment of *Prevotella bivia* in vaginal microbiota, which is tightly regulated by BMI, may be involved in the pathogenesis of SPE.

## Introduction

Severe preeclampsia (SPE) is a hypertensive disorder of pregnancy that can have serious consequences for both maternal and neonatal health^1^. Marked by blood pressure levels > 160/110 mm Hg and other manifestations of a multisystem disorder (e.g., severe proteinuria, thrombocytopenia, impaired liver function, severe persistent right upper quadrant or epigastric pain, renal insufficiency, pulmonary oedema or new-onset headache)^2^, this condition has a complex and only partially understood pathophysiology—with the sole curative treatment being delivery.

In recent years, research on the vaginal microbial community and its role in health and disease has increased considerably^3,4^. Notably, vaginal or placental dysbiosis during pregnancy has been previously associated with an increased risk of preterm delivery^5–7^, preterm rupture of membranes and intrauterine growth restriction^5,8–13^. There has also been ample evidence that human gut microbiota can directly or indirectly play a role in the pathogenesis of cardiovascular diseases and hypertension^14–16^.

Previous studies have successfully investigated the vaginal microbiota of pregnant women with metabolic diseases using high-throughput sequencing of the 16S ribosomal RNA (rRNA) gene^17–19^—an analytical approach that allows for the assessment of bacterial diversity within clinical samples and taxonomical classification of bacterial sequences^20^. However, the question as to whether vaginal dysbiosis may play a role in the pathogenesis of SPE remains unanswered. We therefore designed the current study to compare the vaginal microbiota profiles of Taiwanese women with SPE and with normotensive pregnancies. On the basis of previous research^21^, we also hypothesised that SPE could be associated with higher plasma levels of proinflammatory molecules.

## Results

### General characteristics of the study participants

The general characteristics of the women included in the discovery cohort are summarised in Table 1. Cases with SPE were found to differ significantly from control women with respect to four variables: gestational age (lower in cases with SPE), body mass index (BMI; higher in cases with SPE), betamethasone use for foetal lung immaturity (higher in cases with SPE) and neonatal birth weight (lower in cases with SPE). The general characteristics of the expanded validation cohort are reported in Supplementary Table 1.

**Table 1:** Clinical characteristics of the discovery cohort.

Characteristics	Entire cohort (n = 60)	Control women (n = 30)	Cases with severe preeclampsia (n = 30)	Univariate analysis		Multivariable analysis	
				*P* value	OR (95% CI)	*P* value	OR (95% CI)
*Maternal*							
**GA (weeks)**							
Median (range)	37.0 (28.0–40.0)	38.5 (35.0–40.0)	35.0 (28.0–39.0)	< 0.001**	0.41 (0.26–0.66)	< 0.001**	0.91 (0.89–0.94)
**Age (years)**							
Median (range)	34.5 (24.0–44.0)	34.0 (25.0–41.0)	36.0 (24.0–44.0)	0.762	1.02 (0.92–1.12)		
**BMI (kg/m**^**2**^**)**							
Median (range)	29.2 (20.9–46.6)	26.2 (20.9–40.0)	31.7 (23.8–46.6)	0.001*	1.30 (1.12–1.52)	< 0.001**	1.03 (1.02–1.05)
**Haemoglobin before CS (g/dL)**							
Median (range)	11.5 (6.2–15.0)	11.1 (7.2–14.3)	11.9 (6.2–15.0)	0.474	1.10 (0.84–1.45)		
**Parity**							
Primipara	20 (33.3%)	5 (16.7%)	15 (50.0%)	0.008*	0.20 (0.06–0.66)	0.002*	0.74 (0.62–0.89)
Multipara	40 (66.7%)	25 (83.3%)	15 (50.0%)				
**Betamethasone use (12 mg, 2 doses/day)**							
Yes	11 (18.3%)	0 (0.0%)	11 (36.7%)	0.001^a^*	2.55E + 09 (1.44E + 09 – 4.85E + 09)		
No	49 (81.7%)	30 (100.0%)	19 (63.3%)				
*Newborn*							
**Neonatal birth weight**							
SGA	7 (11.1%)	0 (0.0%)	7 (21.9%)	0.008*			
AGA	50 (79.4%)	29 (93.5%)	21 (65.6%)				
LGA	6 (9.5%)	2 (6.5%)	4 (12.5%)				

^a^Unless otherwise indicated, bootstrap results are based on 1000 bootstrap samples. Data are reported as counts and percentages, unless stated otherwise.

Abbreviations: GA = gestational age; BMI = body mass index; CS = caesarean section; SGA = small for gestational age; AGA = appropriate for gestational age; LGA = large for gestational age; OR = odds ratio; CI = confidence interval.

**P < 0.001.

*P < 0.05.

### Diversity and richness of vaginal microbiota in the study participants

**Figure 1:**
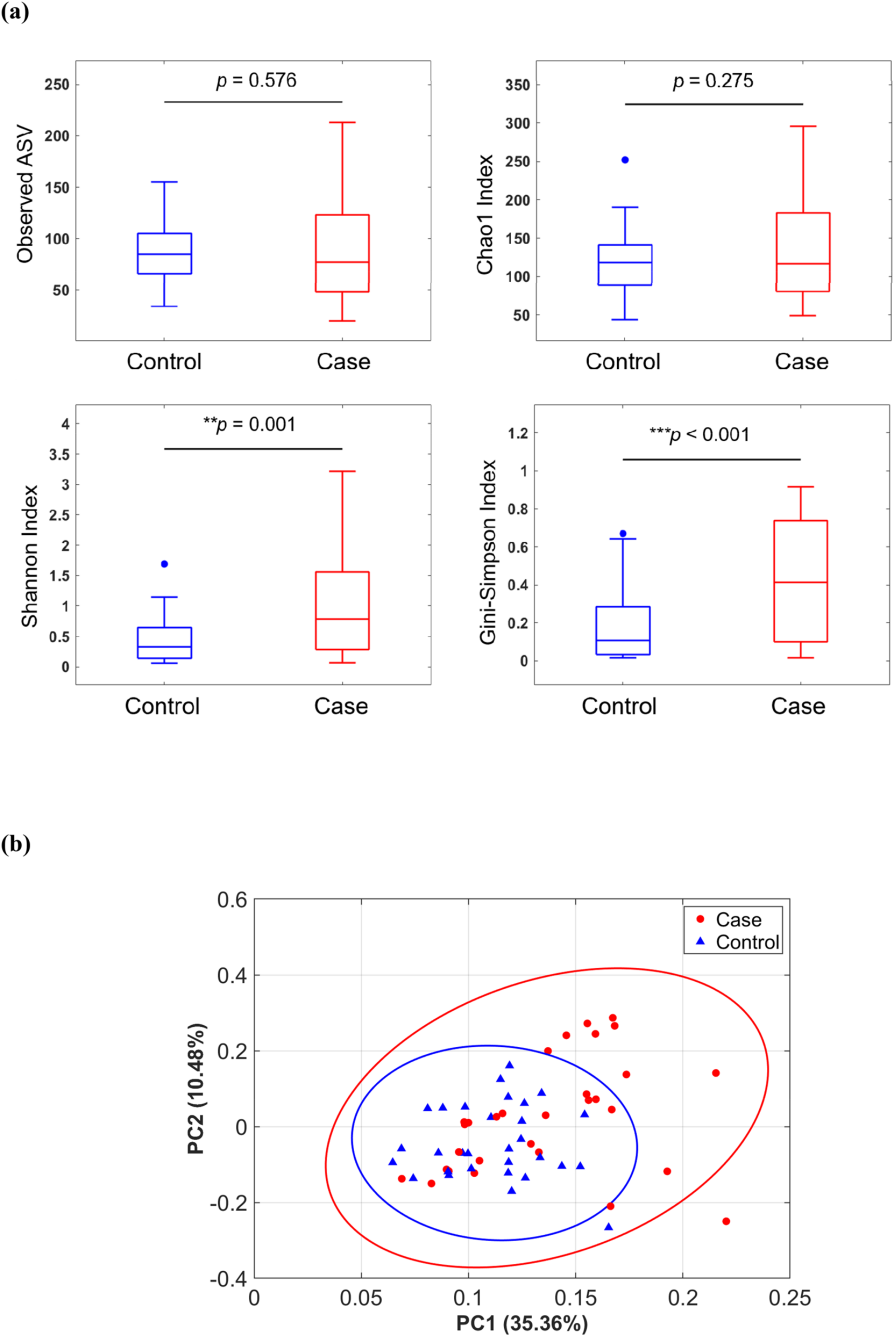
Vaginal microbiota community in cases with severe preeclampsia (n = 30) and control women (n = 30). (**a**) Richness and diversity indices of the vaginal microbiota were calculated from 16S ribosomal RNA gene sequencing of the V3–V4 region on a MiSeq platform. (**b**) A principal component analysis plot was constructed using unweighted UniFrac analysis.

The mean number of raw reads per sample was 2.2 × 10^5^ for all participants. After exclusion of chimera reads and selection of qualified reads, the mean effective number of reads per sample was 1.24 × 10^5^ and 0.90 × 10^5^ for cases with SPE and controls, respectively. The rarefaction curve revealed that the sequencing depth in each sample was sufficient to capture most of the microbial diversity after reaching a saturation plateau phase (data not shown). Species richness in the vaginal microbiota did not differ significantly between cases with SPE and controls. However, both Shannon (*P* = 0.001) and Gini-Simpson (*P* < 0.001) indices were higher in cases with SPE, indicating that their vaginal microbiota was characterised by a greater diversity than that of controls (Fig. 1a). The unweighted principal component analysis (PCA) plot revealed similar vaginal microbiota between the two study groups (Fig. 1b).

### Differences in relative abundances of vaginal bacteria in SPE

**Figure 2:**
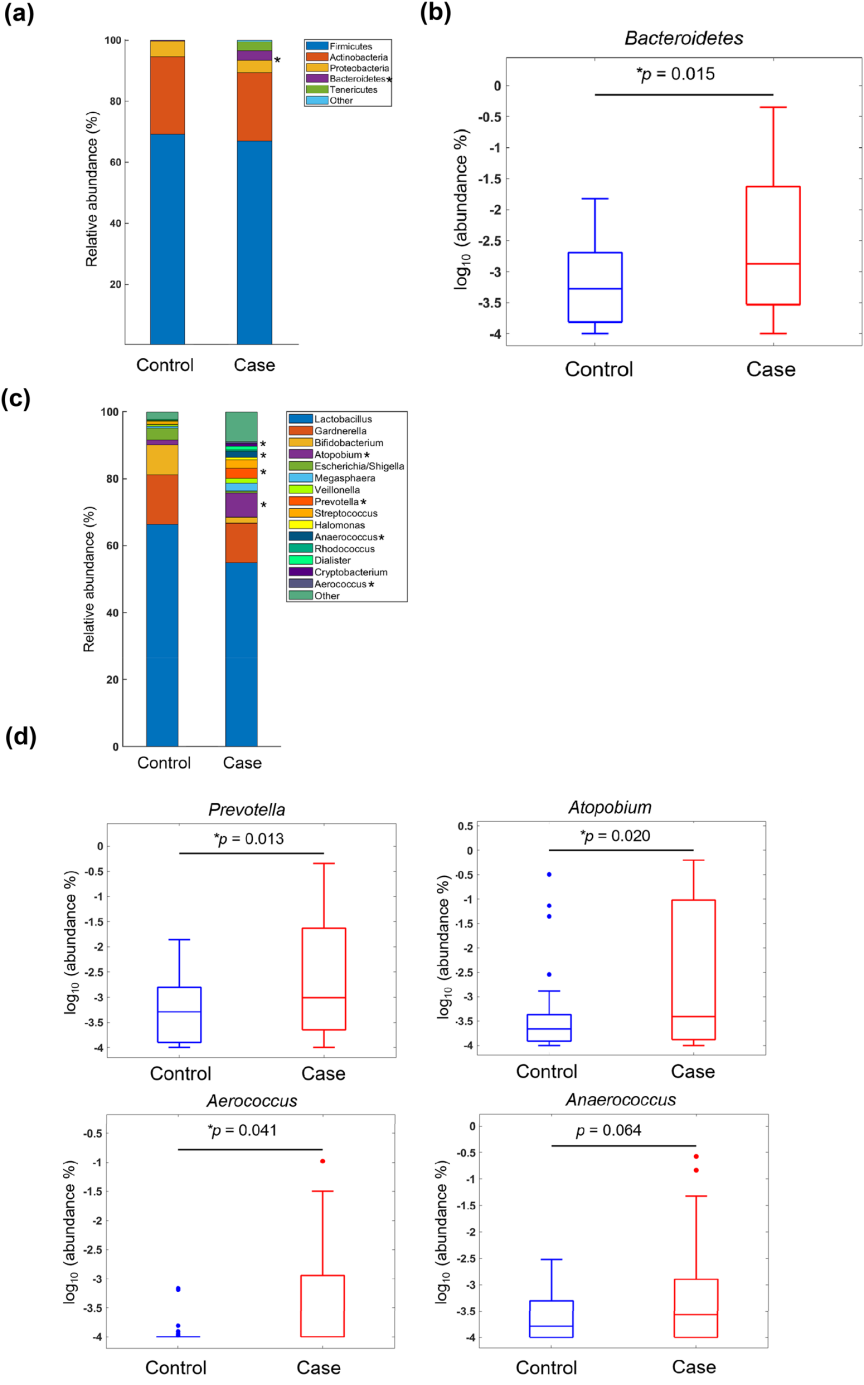
Relative abundance of different bacteria phyla and genera in cases with severe preeclampsia (n = 30) and control women (n = 30). (**a**) The five most abundant bacteria at the phylum level are displayed. (**b**) Box plots indicate a statistically significant difference in the relative abundance of Bacteroidetes between cases with severe preeclampsia and control women. (**c**) The 15 most abundant bacteria at the genus level are displayed. (**d**) Box plots indicate statistically significant differences in the relative abundances of *Prevotella*, *Atopobium*, *Anaerococcus* and *Aerococcus* between cases with severe preeclampsia and control women.

**Figure 3:**
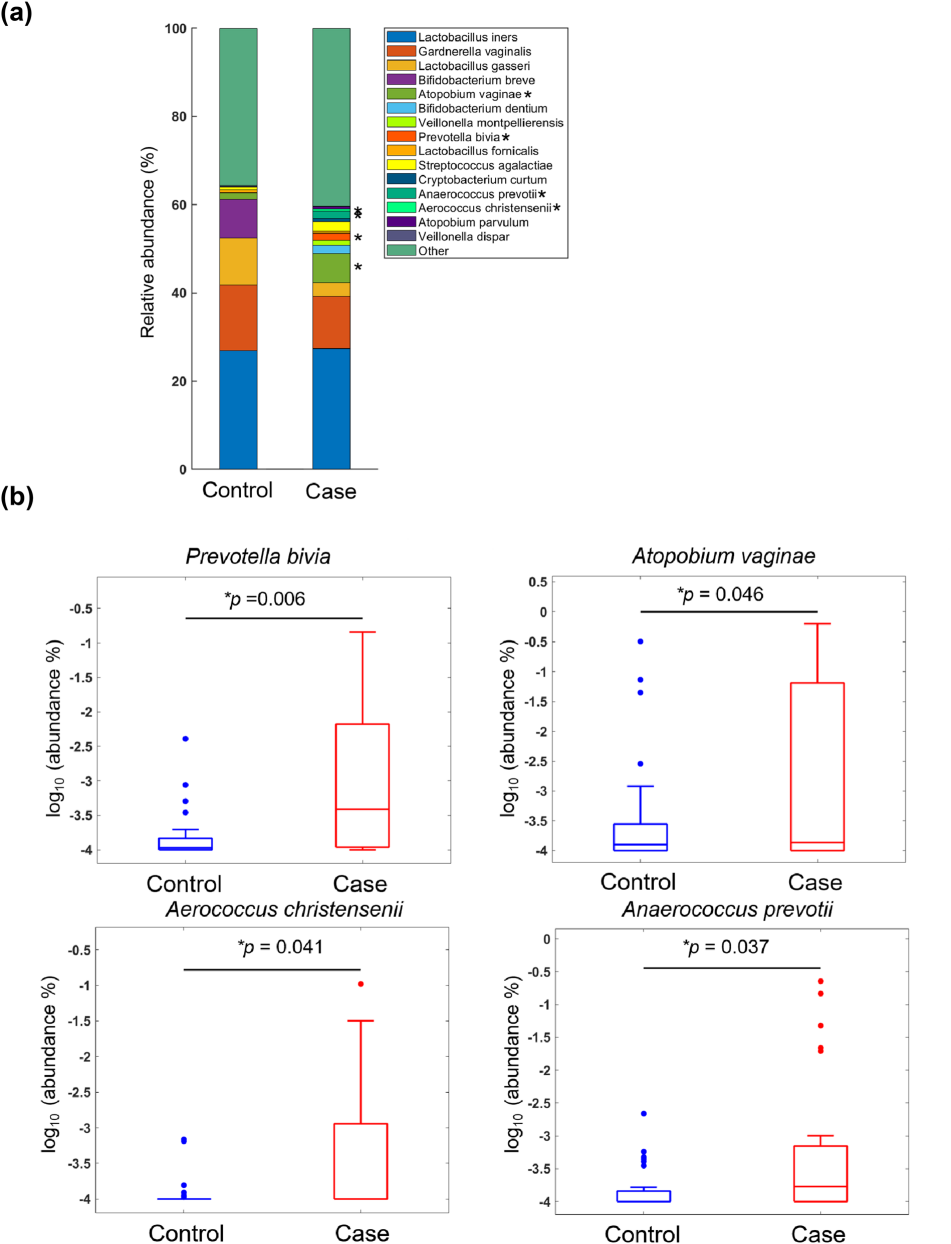
Relative abundance of different bacteria species in cases with severe preeclampsia (n = 30) and control women (n = 30). (**a**) The 15 most abundant bacteria at the species level are displayed. (**b**) Box plots indicate statistically significant differences in the relative abundances of *Prevotella bivia*, *Atopobium vaginae*, *Aerococcus christensenii* and *Anaerococcus tetradius* between cases with severe preeclampsia and control women.

**Figure 4:**
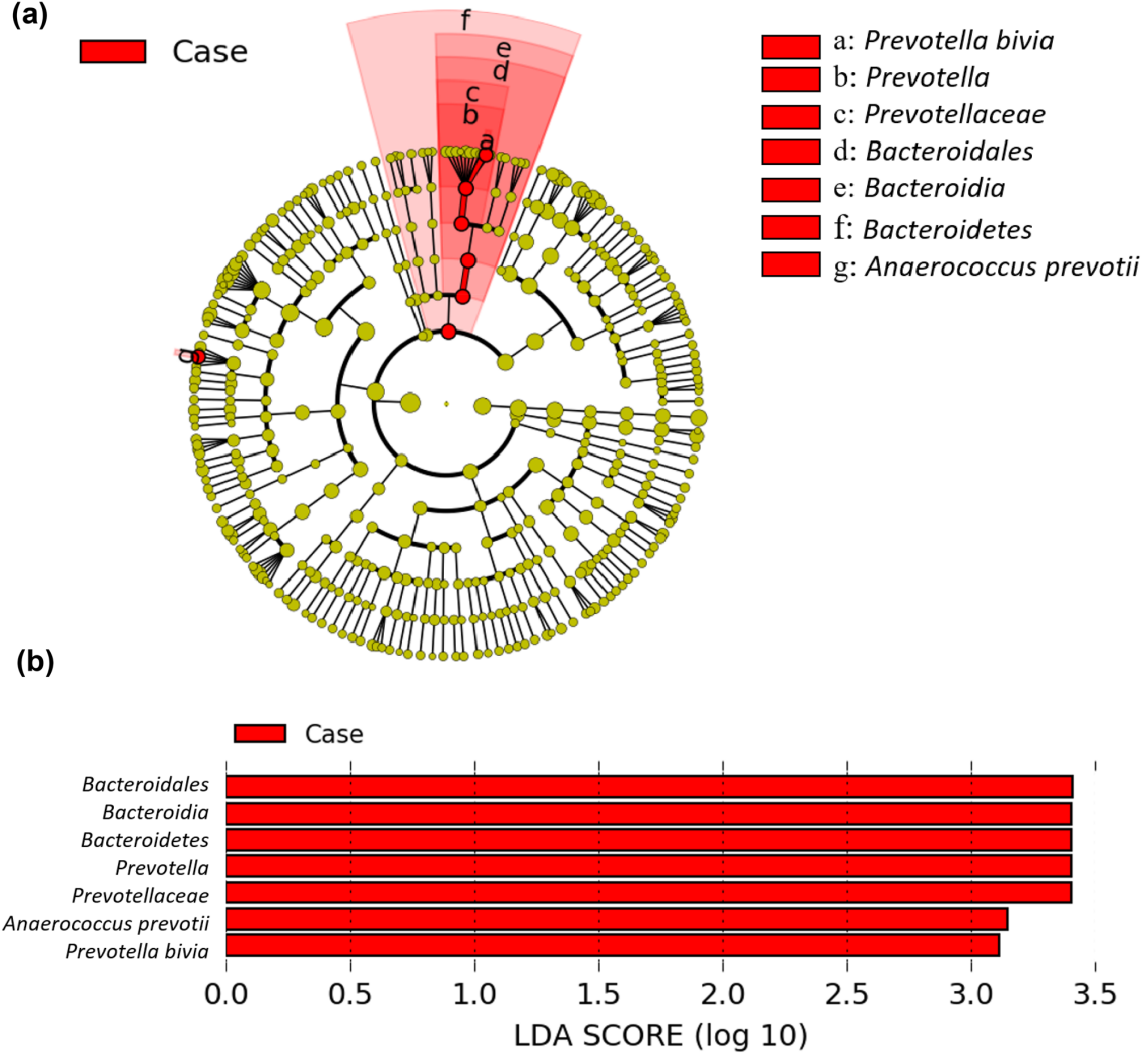
Differences in taxonomic distributions associated with the presence of severe preeclampsia according to a linear discriminant analysis (LDA) of effect size (LEfSe) model, https://bitbucket.org/biobakery/biobakery/wiki/lefse, bioBakery V1.8. (**a**) Bacterial taxa showing different abundances between cases with severe preeclampsia and control women were visualized using a cladogram generated from the LEfSe analysis. (**b**) A log LDA score above 3.00 indicated an increased abundance of amplicon sequence variants (ASVs) attributable to Bacteroidetes, Bacteriodia, Bacteroidales, *Prevotella*, Prevotellaceae and *Prevotella bivia* in cases with SPE.

The relative abundance of bacteria was analysed in the two study groups at the main taxonomic ranks (phylum, genus and species) using gene sequencing of the V3–V4 region on a MiSeq platform. The five most common phyla identified in the vaginal microbiota of control women were Firmicutes (69.12%), Actinobacteria (25.44%), Proteobacteria (5.18%), Bacteroidetes (0.18%) and Tenericutes (0.07%), which altogether accounted for 99% of all detected bacteria (Fig. 2a). Notably, the relative abundance of Bacteroidetes was significantly higher in cases with SPE (3.13%) than in control women (0.18%; *P* = 0.015; Fig. 2b), although no differences were observed with respect to the remaining four phyla. As far as the genus rank is concerned, the 15 dominant genera (> 1%) were *Lactobacillus*, *Gardnerella*, *Bifidobacterium*, *Atopobium*, *Escherichia/Shigella*, *Veillonella*, *Prevotella*, *Streptococcus*, *Klebsiella*, *Halomonas*, *Rhodococcus*, *Dialister*, *Anaerococcus*, *Cryptobacterium* and *Aerococcus* (Fig. 2c). Among them, we found that the relative abundances of *Prevotella* (*P* = 0.013), *Atopobium* (*P* = 0.020) and *Aerococcus* (*P* = 0.041) were significantly higher in cases with SPE than in controls (Fig. 2d). A similar—albeit not statistically significant—trend was observed for *Anaerococcus* (*P* = 0.064). When the four genera were subjected to multivariable logistic regression analysis, only *Prevotella* maintained a significant independent association with SPE (*P* = 0.008; Table 2). We subsequently analysed the distribution of the 15 most common bacterial species in the two study groups (Fig. 3a). Compared with controls, cases with SPE showed a higher relative abundance of *Prevotella bivia* (*P* = 0.006), *Atopobium vaginae* (*P* = 0.046), *Aerococcus christensenii* (*P* = 0.041) and *Anaerococcus tetradius* (*P* = 0.037, Fig. 3b). Multivariable logistic regression analysis revealed that *Prevotella bivia* was the only bacterial species that showed an independent association with SPE (*P* < 0.001; Table 3). Finally, we conducted a linear discriminant analysis (LDA) of effect size (LEfSe) to identify the differences in taxonomic distributions between cases with SPE and control women. SPE samples were found to have significantly higher levels of *Prevotella bivia* and *Anaerococcus prevotii* (Fig. 4, marked with a and f). Moreover, the results confirmed that the *Prevotella bivia* species, *Prevotellaceae* genus, Prevotella family, Bacteroidales order, Bacteriodia class and Bacteroidetes phylum (Fig. 4A, marked with a, b, c, d, and e, respectively) were significantly enriched in the vaginal microbiota of cases with SPE. Collectively, these findings are in agreement with the above observations that link vaginal microbiota composition to the pathogenesis of SPE through the enrichment of *Prevotella bivia*.

**Table 2:** Bacteria genera identified in the discovery cohort (n = 60).

Genera	Control women (n = 30)	Cases with severe preeclampsia (n = 30)	Univariate analysis		Multivariable analysis	
			*P* value	OR (95% CI)	*P* value	OR (95% CI)
*Prevotella*^*a*^	− 3.29 ± 0.61	− 2.66 ± 1.10	0.013*	2.29 (1.19–4.41)	0.008*	1.20 (1.05–1.37)
*Atopobium*^*a*^	− 3.38 ± 0.88	− 2.59 ± 1.44	0.020*	1.75 (1.09–2.80)	–	–
*Aerococcus*^*a*^	− 3.93 ± 0.21	− 3.45 ± 0.96	0.041*	4.14 (1.06–16.17)	–	–
*Anaerococcus*^*a*^	− 3.61 ± 0.44	− 3.20 ± 1.02	0.064	2.13 (0.96–4.75)	–	–

^a^Data are presented as mean log10 (abundance [%] ± standard deviation). Abbreviations: OR = odds ratio; CI = confidence interval.

*P < 0.05.

**Table 3:** Bacteria species identified in the discovery cohort and validation cohort.

Bacteria species	Control women (n = 30)	Cases with severe preeclampsia (n = 30)	Univariate analysis		Multivariable analysis	
			*P* value	OR (95% CI)	*P* value	OR (95% CI)
**16S rRNA gene sequencing (discovery cohort; n = 60)**						
*Prevotella bivia*^*a*^	− 3.49 ± 0.92	− 2.82 ± 1.48	0.006*	6.01 (1.68–21.44)	< 0.001**	1.30 (1.15–1.47)
*Atopobium vaginae*^*a*^	− 3.83 ± 0.35	− 2.96 ± 1.10	0.046*	1.58 (1.01–2.49)	–	–
*Aerococcus christensenii*^*a*^	− 3.93 ± 0.21	− 3.45 ± 0.96	0.041*	4.14 (1.06–16.17)	–	–
*Anaerococcus prevotii*^*a*^	− 3.82 ± 0.33	− 3.31 ± 1.02	0.037*	3.55 (1.08–11.65)	–	–
**Real-time qPCR (validation cohort; n = 113)**						
*Prevotella bivia*^*b*^	− 11.99 ± 5.05	− 8.83 ± 7.29	0.011*	1.08 (1.02–1.15)	0.009*	1.02 (1.01–1.03)
*Atopobium vaginae*^*b*^	− 10.08 ± 5.94	− 8.16 ± 7.79	0.145	1.04 (0.99–1.10)	–	–

^*a*^Data are presented as mean log10 (abundance [%] ± standard deviation). ^*b*^Data are displayed as mean −∆∆Ct ± standard deviation. Abbreviations: OR = odds ratio; CI = confidence interval.

**P < 0.001, *P < 0.05.

### Validation of sequencing results by real-time qPCR

**Figure 5:**
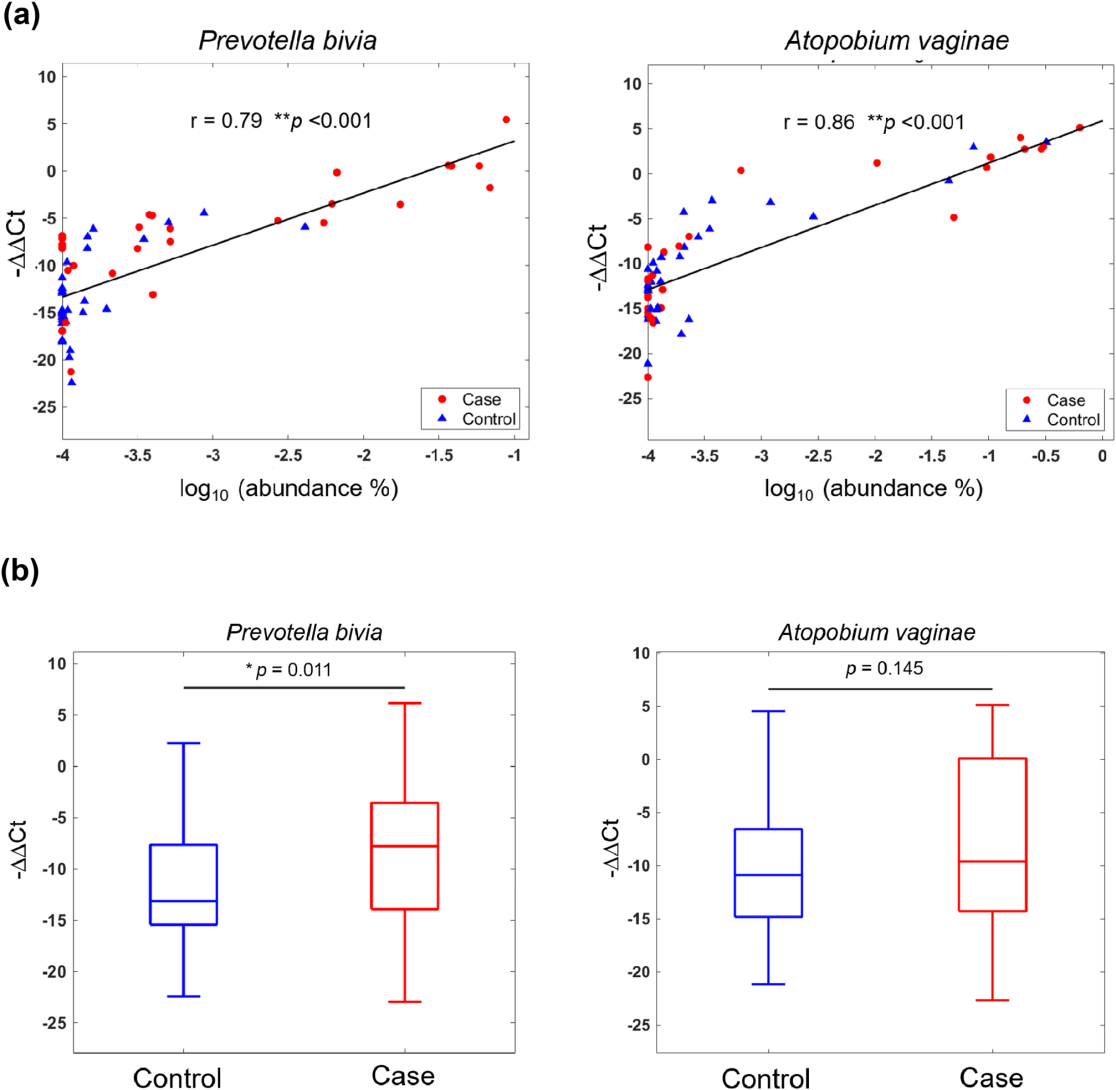
Extent of vaginal colonization by *Prevotella bivia* and *Atopobium vaginae*: correlation analysis between the results of 16S ribosomal RNA gene sequencing and real-time qPCR. (**a**) In the discovery cohort, the relative abundance of both *Prevotella bivia* (r = 0.79, *P* < 0.001) and *Atopobium vaginae* (r = 0.86, *P* < 0.001) as determined by real-time qPCR showed a significant positive association with the results obtained using gene sequencing. (**b**) Based on the results of real-time qPCR, box plots indicate a statistically significant difference in the relative abundance of *Prevotella bivia—*but not of *Atopobium vaginae—*in the extended validation cohort (58 cases with severe preeclampsia and 55 control women).

We sought to validate the results pertaining to the two bacterial species showing the highest relative abundances in cases with SPE versus control women (*Prevotella bivia* and *Atopobium vaginae*) using real-time qPCR. In the discovery cohort, the relative abundance of both *Prevotella bivia* (r = 0.79, *P* < 0.001; Fig. 5a) and *Atopobium vaginae* (r = 0.86, *P* < 0.001) as determined by qPCR showed a significant positive association with the results obtained using gene sequencing. We therefore applied real-time qPCR to investigate the relative abundance of the two bacterial species in the expanded validation cohort. The results confirmed a higher relative abundance of *Prevotella bivia* in the vaginal microbiota of cases with SPE (*P* = 0.011), although significant differences were no longer evident for *Atopobium vaginae* (Fig. 5b). Also, *Prevotella bivia* showed an independent association with SPE (*P* = 0.009) in the validation cohort by multivariable logistic regression analysis (Table 3).

### Plasma cytokine levels

**Figure 6:**
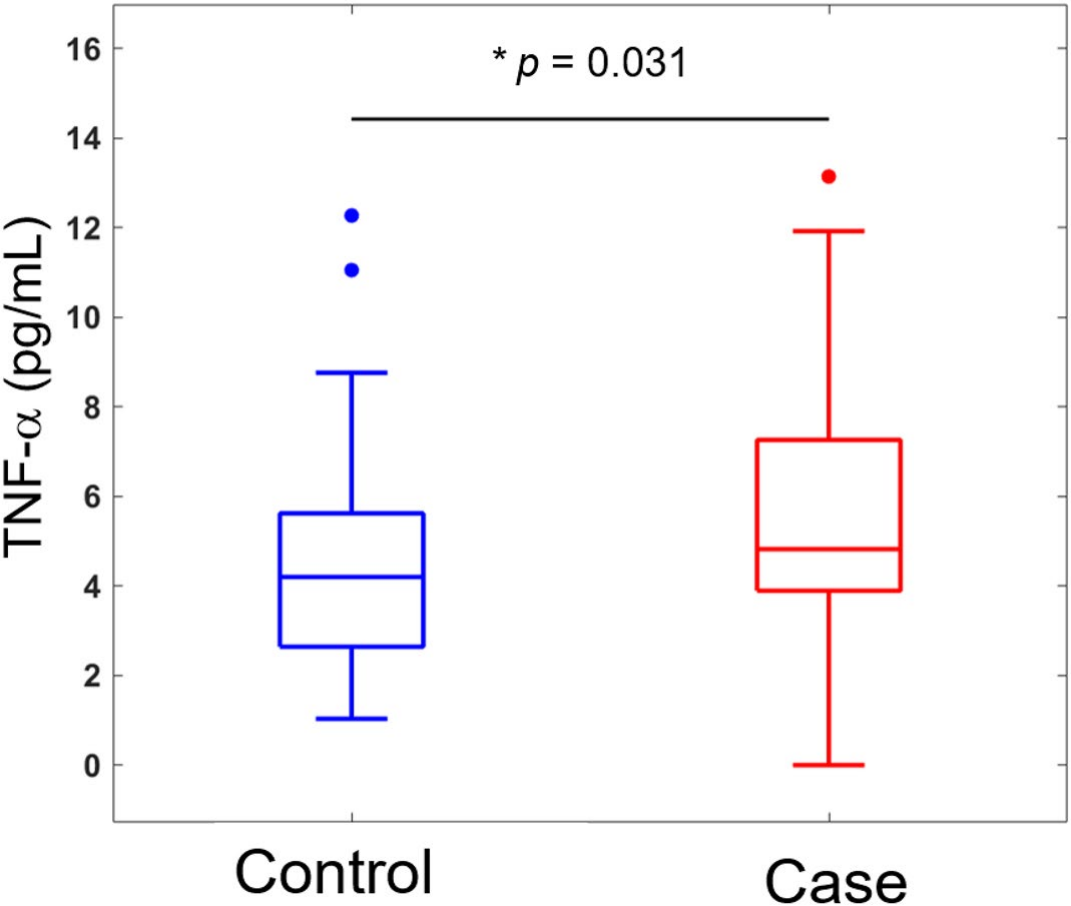
The results of a multiplex ELISA-based chemiluminescent assay revealed an increase in plasma TNF-α levels for women with severe preeclampsia compared with control women.

Data from the multiplex ELISA-based chemiluminescent assay conducted in the expanded validation cohort revealed that plasma levels of tumour necrosis factor alpha (TNF-α)—but not of other cytokines—were significantly higher in cases with SPE compared with control women (*P* = 0.031). This difference was consistently observed in both univariate and multivariable logistic regression analyses (Fig. 6; Supplementary Table 2).

### Three-variable panel to discriminate between SPE and normotensive uncomplicated pregnancies

Of the different variables under study, three (vaginal *Prevotella bivia*, plasma TNF-α and BMI) were included in a prediction panel for SPE (Table 4). The area under the receiver operating characteristic curve (AUC) of predicted probability values for the three-variable panel revealed that it can discriminate between SPE and normotensive pregnancies with slightly better accuracy than BMI, *Prevotella bivia* and TNF-α alone (AUC = 0.797, sensitivity = 0.655; specificity = 0.764; accuracy = 0.708; Supplementary Fig. 1).

**Table 4:** Characteristics of different variables and multimarker panels for distinguishing between cases with SPE and control women.

Variables	Sensitivity	Specificity	Accuracy	AUC (95% CI)
BMI	0.655	0.764	0.708	0.772 (0.674–0.851)
*Prevotella bivia*	0.655	0.655	0.655	0.637 (0.530–0.733)
*Prevotella bivia* + BMI	0.672	0.709	0.69	0.785 (0.691–0.862)
TNF-α	0.534	0.618	0.575	0.631 (0.526–0.735)
TNF-α + BMI	0.690	0.709	0.699	0.790 (0.696–0.861)
*Prevotella bivia* + TNF-α	0.569	0.673	0.619	0.665 (0.555–0.754)
*Prevotella bivia* + TNF-α + BMI	0.655	0.764	0.708	0.797 (0.705–0.864)

The highest AUC was observed for the three-variable panel consisting of vaginal *Prevotella bivia*, plasma TNF-α and BMI. Abbreviations: AUC = area under curve; CI = confidence interval; BMI = body mass index; TNF-α = tumour necrosis factor alpha.

To remove the confounding factor of gestational diabetes mellitus, we performed a subgroup analysis excluding 20 women with concomitant gestational diabetes from the group of women with SPE. The higher relative abundance of *Prevotella bivia*, elevated TNF-α levels and higher BMI in SPE as well as the model of prediction combining *Prevotella bivia*, TNF-α and BMI remained valid for discriminating between SPE and normotensive pregnancies (Supplementary Table 3).

## Discussion

The main results of our study indicate that women with SPE have a higher relative abundance of *Prevotella bivia* in their vaginal microbiota and higher plasma levels of the proinflammatory cytokine TNF-α—the latter finding being in line with the published literature^22^. Since obese women have a higher risk of preeclampsia, BMI is one of the most important predictive variables. A logistic regression model adjusted for BMI was constructed on log-transformed data to identify differences in taxonomic distributions. After adjusting for BMI, the associations of *Atopobium vaginae*, *Aerococcus christensenii* and *Anaerococcus tetradius* with the disease were removed. Only *Prevotella bivia* retained independent significance for the prediction of SPE in the discovery cohort. However, the higher relative abundance of *Prevotella bivia* in women with SPE was only marginally significant after adjustment for BMI (*P* = 0.07) in the confirmation cohort, suggesting that the role of *Prevotella bivia* in SPE may be modified by BMI. We further combined these two parameters with maternal BMI to devise a three-variable panel that discriminates between SPE and normotensive pregnancies with good accuracy.

*Prevotella bivia* is an anaerobic gram-negative bacterial species that has been previously associated with pelvic inflammatory disease and bacterial vaginosis^23,24^. A shift from the physiological lactobacilli-dominated vaginal microbiota to a relative abundance of anaerobic and facultative anaerobic bacteria has been shown to play a role in different adverse obstetric and gynaecologic outcomes, including miscarriage, premature labour, preterm birth, preterm premature rupture of membranes, chorioamnionitis, intrauterine infection, post-caesarean endometritis, upper genital tract infections and pelvic inflammatory disease^3,7,10,12,25,26^. Interestingly, inflammation of the chorionic plate has been associated with the presence of several bacterial species in the preeclamptic placenta—including *Bacillus cereus*, *Listeria*, *Salmonella*, *Escherichia*, *Klebsiella pneumonia*, *Anoxybacillus*, *Variovorax*, *Prevotella*, *Porphyromonas* and *Dialister*^27^. The question as to whether bacteria are capable of ascending from the vagina to the chorionic plate during the course of preeclamptic pregnancies remains unanswered and requires further scrutiny.

Previous research demonstrated that obesity is a risk factor for preeclampsia^28^ and that the vaginal microbial community of obese women is characterised by an increased diversity with a relative preponderance of *Prevotella* spp.^17^ Here, we show that both BMI and a higher relative abundance of *Prevotella bivia* in the vaginal microbiota were associated with SPE. Notably, the preponderance of *Prevotella oralis* in the oral microbiome has been previously associated with blood pressure outcomes in older women^29^. It is thus possible that the *Prevotella* genus may play a role not only in the pathogenesis of hypertension in general but also in hypertensive disorders of pregnancy. In our study, vaginal swab samples used for microbiota analysis were obtained at the time of caesarean section—a procedure which has been related to a lower abundance of Bacteroidetes in the intestinal microbiota of infants^30^. Although a previous study demonstrated that maternal overweight and obesity do not affect infant gut microbiota composition and diversity in caesarean-delivered infants^31^. we believe that further research on the associated between preeclampsia, obesity, maternal vaginal microbiota and infant gut microbiota after caesarean section is warranted. Accordingly, no information on the occurrence of preeclampsia was provided by Singh et al.^31^.

Preeclampsia is accompanied by a systemic inflammatory response^32^, and the ischemic placental injury occurring in this condition has been associated with an increased release of TNF-α in the maternal bloodstream^33^. Using a multiplex assay for eight different cytokines, TNF-α was the only inflammatory biomarker found to be increased in the plasma of our cases with SPE. Notably, we devised a three-variable panel—comprising BMI, vaginal *Prevotella bivia* and plasma TNF-α—that is capable of discriminating between SPE and normotensive uncomplicated pregnancies with good accuracy. The complex relationships between the three variables deserve further scrutiny in independent investigations.

Several limitations of our study merit comment. First, the sample size was small and our results should be considered as preliminary and hypothesis-generating. All of the study participants were of Taiwanese descent, potentially limiting the generalizability of our findings. Second, we did not specifically investigate whether the maternal vaginal microbiome at the time of caesarean delivery could affect the gut microbiota of newborns. Third, we did not specifically screen for common genital infections—including bacterial vaginosis, vulvovaginal candidiasis, and *Chlamydia trachomatis* infection. Nonetheless, all participants had national health insurance to cover human immunodeficiency virus and syphilis screening at the first prenatal appointment. Moreover, all newborns tested negative for *Chlamydia trachomatis* on urine samples collected immediately after delivery. There was no evidence of clinical vaginitis with profuse abnormal discharge suggestive of either bacterial vaginosis or candidiasis when collecting the vaginal swabs. Despite these limitations, we believe that our study may pave the way for clinical testing for *Prevotella bivia* vaginal colonization during pregnancy. Moreover, this assay may be used to determine whether a relative abundance of *Prevotella bivia* in vaginal microbiota along with BMI and plasma TNF-α during pregnancy can more accurately predict future development of SPE.

In summary, our results indicate that the higher relative abundance of *Prevotella bivia* in the vaginal microbial community, which is related to increased BMI, may be involved in the pathogenesis of SPE. Our findings may prompt further studies with a larger sample size on *Prevotella bivia* colonization in the vagina, with the ultimate goal of reducing hypertensive complications of pregnancy.

## Materials and methods

### Design, participants and study variables

This single-centre, prospective cohort study was conducted at the Perinatal Care Clinic and Delivery Room of the Chang Gung Memorial Hospital (Linkou, Taiwan) between November 2017 and June 2019. Women with SPE were recruited during the third trimester of pregnancy after 28 weeks of gestation. Clinical management of SPE followed the American College of Obstetricians and Gynecologists guidelines^2^ and consisted of MgSO_4_ administration to prevent seizure attacks, steroid use to improve foetal lung maturity (when the gestational age was < 34 weeks), anti-hypertensive drugs to reduce blood pressure and electronic foetal heartbeat tracing. Prenatal antibiotics were not used. The control group consisted of women with normotensive term pregnancies who were scheduled for caesarean section (because of a history of previous caesarean section or foetal malpresentation). Preterm delivery was defined as delivery before 37 weeks of gestation. Cases with a gestational age of less than 28 weeks, smoking or chronic illnesses (e.g., asthma, systemic lupus erythematosus or rheumatoid arthritis) were excluded. The general characteristics of mothers and children were collected from clinical and birth records. All participants were of Taiwanese descent. According to the criteria of the National Health Bureau of Taiwan, BMI was categorized as follows: normal (< 24 kg/m^2^), overweight (24–27 kg/m^2^) and obese (> 27 kg/m^2^). Newborn birth weight was classified into the following categories: small for gestational age (SGA; birth weight below the 10th percentile), appropriate for gestational age (AGA; birth weight from 10 to 90th percentile) and large for gestational age (LGA; birth weight above the 90th percentile)^1^. The discovery cohort consisted of 30 women with SPE (eight with concomitant gestational diabetes) and 30 control women. We sought confirmation of our findings in an expanded cohort consisting of 58 women with SPE (20 with concomitant gestational diabetes) and 55 controls. The study followed the tenets of the Declaration of Helsinki and was granted ethical approval by the Chang Gung Memorial Hospital Institutional Review Board (approval number: 201701371A3). Informed consent was obtained from all participants.

### Vaginal swab collection and extraction of bacterial DNA

Vaginal swab specimens were collected from all participants at the time of caesarean section using a commercially available kit (LIBO Medical Products Inc., Taipei, Taiwan). Bacterial DNA for vaginal microbiota analysis was extracted with the QiaAmp DNA Microbiome Kit (Qiagen, Hilden, Germany), washed, eluted with nuclease-free water and stored at − 80 °C. The concentration and quality of purified DNA were determined using the Qubit dsDNA High Sensitivity Assay (Thermo Fisher Scientific, Waltham, MA, USA).

### Sample preparation, library construction and sequencing

We initially constructed a 16S rRNA gene amplicon library targeting the 16S rRNA V3–V4 region. Illumina adapter overhang nucleotide sequences were subsequently added to gene-specific sequences. The 16S rRNA gene amplicon PCR primers were as follows: forward, 5′-TCGTCGGCAGCGTCAGATGTGTATAAGAGACAGCCTACGGGNGGCWGCAG-3′, and reverse, 5′-GTCTCGTGGGCTCGGAGATGTGTATAAGAGACAGGACTACHVGGGTATCTAATCC-3′. The initial PCR mixture consisted of bacterial DNA (10 ng), forward and reverse primers (1 μM each) and 1 × KAPA HiFi Hotstart ReadyMix (Kapa Biosystems, Wilmington, MA, USA). Conditions for the first PCR were as follows: an initial denaturation at 95 °C for 3 min followed by 25 cycles of denaturation at 95 °C for 30 s, annealing at 55 °C for 30 s and extension at 72 °C for 30 s followed by a final extension at 72 °C for 30 s. PCR products were purified using AMPure XP beads (Beckman Coulter, Brea, CA, USA) and subsequently subjected to index PCR. The index PCR mixture consisted of the purified products of the first PCR, Nextera XT index primers 1 and 2 (5 μL each; Illumina, San Diego, CA, USA) and 1 × KAPA HiFi Hotstart ReadyMix. The following conditions were used for the index PCR: initial denaturation at 95 °C for 3 min followed by eight cycles of denaturation at 95 °C for 30 s, annealing at 55 °C for 30 s and extension at 72 °C for 30 s followed by a final extension at 72 °C for 5 min. The final amplicon libraries (approximate length: 630 bp) were validated using the HT DNA High Sensitivity LabChip Kit (Caliper; Perkin-Elmer, Shelton, CT, USA). Multiplexed pooled libraries were sequenced on a MiSeq system with 2 × 300 paired-end v3 sequencing reagents (Illumina). The raw sequence files of 16 s supporting the findings of this article are available in the NCBI Sequence Read Archive under the BioProject ID PRJNA663041 https://dataview.ncbi.nlm.nih.gov/object/PRJNA663041?reviewer=9o8df9kefddea8p6feqta4kvtg .

### Bioinformatics analysis of amplicon library sequences

Sequencing reads were initially de-multiplexed using the MiSeq Reporter software v2.6 (Illumina) based on sample barcodes. The Usearch tool v11 (https://drive5.com/) was used to perform raw reads. In brief, pair reads were joined, and quality filtering for expected errors was implemented (maximum threshold: 1.0). Samples with < 60,000 merged reads were excluded from the analysis. In each dataset, the Cutadapt v1.14 package^34^ was used to remove forward and reverse sequencing primers from the merged reads. Merged sequences < 400 bp in length were not included in the analysis. Denoising of effective reads from de-replication reads was conducted using the UNOISE tool^35^ with the goal of identifying all correct biological sequences in the zero-radius operational taxonomic unit, i.e., amplicon sequence variants (ASVs). The final taxonomic assignment was performed using the SINTAX classifier^36^. Species richness and diversity of the vaginal microbiota were determined from the number of bacterial species assigned by the ASVs. Specifically, richness was estimated with the Observed ASV and Chao1 indices, whereas diversity was determined by calculating the Shannon index and the Gini-Simpson index. Finally, LEfSe^20^ was used to identify the taxonomy of vaginal bacteria that were most likely to distinguish between cases of SPE and controls.

### Real-time qPCR

Extraction of genomic bacterial DNA was performed with the QiaAmp DNA Microbiome Kit (Qiagen) according to the manufacturer’s instructions. The TaqMan gene expression assay (Applied Biosystems, Foster City, CA, USA) was used to analyze the expression levels of DNA from *Prevotella bivia* (Taqman: Ba04646278_s1) and *Atopobium vaginae* (Ba04646222_s1), with 16S rRNA expression (Taqman: Ba04930791_s1) as an internal control^23^. When no increase in fluorescent signal was evident until cycle 40, the sample was arbitrarily assumed to have a value of 40. The -ΔΔCt was calculated as follows: ΔCt (bacteria) − ΔCt (internal control). The correlation between ΔCt values was also determined.

### Quantification of plasma cytokine levels

Whole-blood specimens were collected from all participants at the time of caesarean section and immediately centrifuged to obtain plasma, which was stored in aliquots (1 mL) at − 80 °C until analysis. Plasma concentrations of eight different cytokines (IL-2, IL-4, IL-6, IL-8, IL-10, GM-CSF, IFN-γ and TNF-α) were determined in the expanded validation cohort using a multiplex ELISA-based chemiluminescent assay (Human Cytokine Group I, 8-plex; Bio-Rad Laboratories Inc., Hercules, CA, USA) according to the manufacturer’s instructions.

### Data analysis

The general characteristics and the biochemical data of the study participants are presented using descriptive statistics and compared with non-parametric tests. Univariate and multivariable analyses of variables associated with SPE were conducted using the SPSS statistical software (version 22.0; IBM, Armonk, NY, USA). The ‘ASVs relative abundances’ were log-transformed to improve normality^37^. Variables independently associated with SPE were used to devise a model to discriminate between SPE and normotensive pregnancies. The sensitivity, specificity, accuracy and AUC of predicted probability values for the panel were calculated using the MATLAB package (2015a) with the integrated ‘perfcurve’ function. Other analyses were conducted in the R environment (https://www.r-project.org/). We performed a logistical regression adjusted for BMI on log-transformed data to identify differences in taxonomic distributions.

## Supplementary information

Supplementary Information
