# Supplementary material for: Severe preeclampsia is associated with a higher relative abundance of Prevotella bivia in the vaginal microbiota
Source: Sci Rep. 2020 Oct 26;10:18249. doi: 10.1038/s41598-020-75534-3 (PMC7588441; doi:10.1038/s41598-020-75534-3)
Supplement: Supplementary file 1 — Supplementary Information [file 41598_2020_75534_MOESM1_ESM.doc]

**Severe preeclampsia is associated with a higher relative abundance of *Prevotella bivia* in the vaginal microbiota**

Chia-Ying Lin^1,#^, Chiao-Yun Lin^2,3#^, Yuan-Ming Yeh^4^, Lan-Yan Yang^5^, Yun-Shien Lee^4,6^, Angel Chao^2,3^, Chia-Yin Chin^7^, An-Shine Chao^2,8🞶^, Chia-Yu Yang^7,9,10🞶^

^1^Department of Pediatrics, Chang Gung Memorial Hospital Linkou Medical Center and Chang Gung University College of Medicine, Taoyuan, Taiwan

^2^Department of Obstetrics and Gynecology, Chang Gung Memorial Hospital Linkou Medical Center and Chang Gung University College of Medicine, Taoyuan, Taiwan ^3^Gynecologic Cancer Research Center, Chang Gung Memorial Hospital, Taiwan

^4^Genomic Medicine Core Laboratory, Chang Gung Memorial Hospital, Taiwan

^5^Biostatistics Unit, Clinical Trial Center, Chang Gung Memorial Hospital Linkou Medical Center, Taiwan

^6^Department of Biotechnology, Ming Chuan University, Taoyuan, Taiwan

^7^Molecular Medicine Research Center, Chang Gung University, Taoyuan, Taiwan ^8^New Taipei City Municipal Tucheng Hospital, Taiwan

^9^Department of Microbiology and Immunology, College of Medicine, Chang Gung University, Taoyuan, Taiwan

^10^Department of Otolaryngology – Head and Neck Surgery, Chang Gung Memorial Hospital, Taoyuan, Taiwan

^#^These authors contributed equally to this work.

^*^**Correspondence to:**

An-Shine Chao, MD, E-mail: aschao1295@cgmh.org.tw or Chia-Yu Yang, PhD, E-mail: chiayu-yang@mail.cgu.edu.tw

Department of Obstetrics and Gynecology, Chang Gung Memorial Hospital Linkou Medical Center, 5 Fushin St., Guishan, Taoyuan 333, Taiwan

**Supplementary Table 1**. Clinical characteristics of the validation cohort

| Characteristics | Entire cohort  (n = 113) | Control women  (n = 55) | Cases with severe preeclampsia (n = 58) |
| --- | --- | --- | --- |
| *Maternal* | | |  |
| **GA (weeks)** | |  |  |
| Median (range) | 37.0 (28.0–40.0) | 38.0 (35.0–40.0) | 36.0 (28.0–40.0) |
| **Age (years)** | |  |  |
| Median (range) | 35.0 (22.0–48.0) | 34.0 (25.0–48.0) | 36.0 (22.0–48.0) |
| **BMI (kg/m^2^)** | |  |  |
| Median (range) | 28.2 (20.9–75.9) | 26.6 (20.9–40.0) | 30.8 (21.6–75.9) |
| **Haemoglobin before CS (g/dL)** | | | |
| Median (range) | 11.7 (6.1–15.1) | 11.2 (7.2–15.1) | 12.1 (6.1–15.0) |
| **Parity** |  |  |  |
| Primipara | 44 (38.9%) | 11 (20.0%) | 33 (56.9%) |
| Multipara | 69 (61.1%) | 44 (80.0%) | 25 (43.1%) |
| **Betamethasone use (12 mg, 2 doses/day)** | |  |  |
| Yes | 19 (16.8%) | 1 (1.8%) | 18 (31.0%) |
| No | 94 (83.2%) | 54 (98.2%) | 40 (69.0%) |
| *Newborn* | | |  |
| **Neonatal birth weight** | | |  |
| SGA | 14 (11.3%) | 1 (1.7%) | 13 (20.0%) |
| AGA | 95 (76.6%) | 53 (89.8%) | 42 (64.6%) |
| LGA | 15 (12.1%) | 5 (8.5%) | 10 (15.4%) |

Abbreviations: GA = gestational age; BMI = body mass index; CS = caesarean section; SGA = small for gestational age; AGA = appropriate for gestational age; LGA = large for gestational age; OR = odds ratio; CI = confidence interval.

**Supplementary Table 2**. Plasma levels of different cytokines in the validation cohort

| Cytokines ^a^ | Control women  (n = 55) | Cases with severe preeclampsia  (n = 58) | Univariate analysis | | Multivariable analysis | |
| --- | --- | --- | --- | --- | --- | --- |
|  |  |  | P value | OR (95% CI) | P value | OR (95% CI) |
| TNF-α | 4.47 ± 0.31 | 5.53 ± 0.35 | 0.031* | 1.19 (1.02–1.39) | 0.027* | 1.04 (1.01–1.08) |
| IL-2 | 0.27 ± 0.04 | 0.32 ± 0.04 | 0.413 | 1.64 (0.50–5.41) | - | - |
| IL-4 | 0.05 ± 0.01 | 0.03 ± 0.01 | 0.304 | 0.04 (6.52e-05–20.17) | - | - |
| IL-6 | 9.47 ± 2.66 | 15.84 ± 3.50 | 0.163 | 1.01 (0.99–1.03) | - | - |
| IL-8 | 5.96 ± 2.02 | 5.09 ± 0.73 | 0.682 | 0.99 (0.96–1.03) | - | - |
| IL-10 | 0.31 ± 0.09 | 0.34 ± 0.10 | 0.797 | 1.07 (0.62–1.86) | - | - |
| GM-CSF | 0.03 ± 0.01 | 0.03 ± 0.01 | 0.986 | 1.05 (0.01–130.29) | - | - |
| IFN-γ | 0.33 ± 0.09 | 0.35 ± 0.07 | 0.854 | 1.06 (0.57–1.96) | - | - |

*^a^*The unit for plasma concentrations of all cytokines is pg/mL; results are expressed as means ± standard errors of the mean. Abbreviations: TNF = tumour necrosis factor; IL = interleukin; GM-CSF = granulocyte-macrophage colony-stimulating factor; IFN = interferon; OR = odds ratio; CI = confidence interval.

*P < 0.05

**Supplementary Table 3. Characteristics of different variables and multimarker panels for distinguishing between cases with severe preeclampsia and control women after the exclusion of patients with gestational diabetes**

|  |  | | Univariate analysis | |
| --- | --- | --- | --- | --- |
|  | Control women  (n = 55) | Cases with SPE (n = 38) | P value | OR (95% CI) |
| BMI | 26.6 (20.9–40.0) | 30.0 (21.6–75.9) | 0.002 | 1.20 (1.07–1.34) |
| *Prevotella bivia* | -11.99 ± 5.05 | -9.07± 6.96 | 0.025 | 1.09 (1.01–1.17) |
| TNF-α | 4.47 ± 0.31 | 5.65 ± 0.47 | 0.038 | 1.19 (1.01–1.41) |
| Variables | Sensitivity | Specificity | Accuracy | AUC (95% CI) |
| BMI | 0.474 | 0.855 | 0.699 | 0.708 (0.580–0.801) |
| *Prevotella bivia* | 0.316 | 0.836 | 0.624 | 0.631 (0.504–0.736) |
| *Prevotella bivia* + BMI | 0.474 | 0.891 | 0.720 | 0.723 (0.610–0.826) |
| TNF-α | 0.263 | 0.873 | 0.624 | 0.625 (0.503–0.729) |
| TNF-α + BMI | 0.526 | 0.836 | 0.710 | 0.739 (0.614–0.829) |
| *Prevotella bivia*  + TNF-α | 0.421 | 0.855 | 0.677 | 0.660 (0.526–0.775) |
| *Prevotella bivia* + TNF-α + BMI | 0.526 | 0.891 | 0.742 | 0.745 (0.631–0.840) |

Abbreviations: AUC = area under the curve; BMI = body mass index; CI = confidence interval; OR = odds ratio; TNF = tumour necrosis factor.


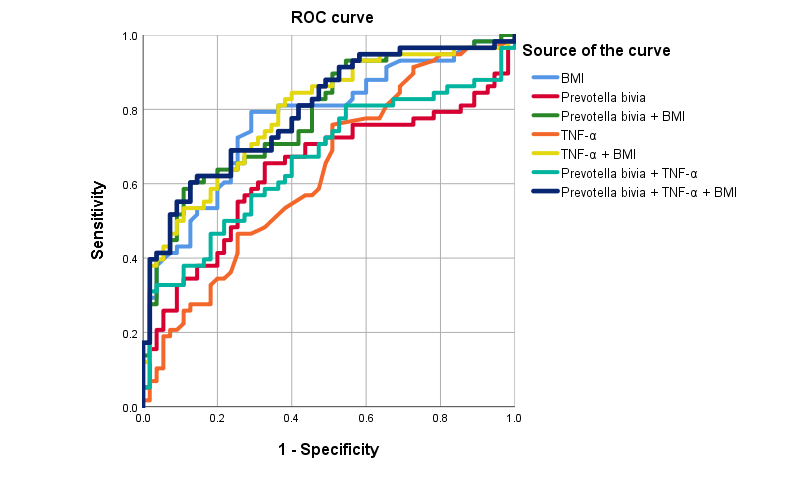


**Supplementary Figure 1.** Receiver operating characteristic curves of different variables and multimarker panels for distinguishing between cases with severe preeclampsia and control women.
